# An Inner Barrier to Career Development: Preconditions of the Impostor Phenomenon and Consequences for Career Development

**DOI:** 10.3389/fpsyg.2016.00048

**Published:** 2016-02-04

**Authors:** Mirjam Neureiter, Eva Traut-Mattausch

**Affiliations:** Economic and Organizational Psychology, Department of Psychology University of SalzburgSalzburg, Austria

**Keywords:** impostor phenomenon, fear of failure, fear of success, self-esteem, career planning, career striving, motivation to lead

## Abstract

The impostor phenomenon (IP) is increasingly recognized as an important psychological construct for career development, yet empirical research on how it functions in this domain is sparse. We investigated in what way impostor feelings are related to the fear of failure, fear of success, self-esteem, and the career-development aspects career planning, career striving, and the motivation to lead. We conducted two studies with independent samples of university students (*N* = 212) in a laboratory study and working professionals (*N* = 110) in an online study. In both samples, impostor feelings were fostered by fear of failure, fear of success, and low self-esteem and they decreased career planning, career striving, and the motivation to lead. A path analysis showed that impostor feelings had the most negative effects on career planning and career striving in students and on the motivation to lead in working professionals. The results suggest that the IP is relevant to career development in different ways at different career stages. Practical implications and interventions to reduce the negative effects of impostor feelings on career development are discussed.

## Introduction

The impostor phenomenon (IP) is defined as an internal experience of intellectual and professional incapability despite objective evidence to the contrary (Clance and Imes, 1978). People who suffer from this phenomenon believe that their success is due to some kind of luck or error, and they live in constant fear of being unmasked as unintelligent or less capable (Clance, 1985; Harvey and Katz, 1986; Jöstl et al., 2012). It has been suggested that about 70% of people from all walks of life feel like impostors for at least some part of their careers (Gravois, 2007). However, the role of the IP in the context of career development remains unclear. Do impostors have very clear career plans to handle their insecurity? Or do impostor fears hold IP sufferers back from striving for higher career stages or positions with increasing responsibility and power?

Many studies have examined the relation of the phenomenon and various clinical variables, such as depression and (social) anxiety (Chrisman et al., 1995; Henning et al., 1998; Thompson et al., 1998; September et al., 2001; Bernard et al., 2002; Oriel et al., 2004; McGregor et al., 2008). Yet despite emerging recognition of the importance of impostor feelings in different cultures (Chae et al., 1995; Clance et al., 1995) and different groups, such as marketing managers (Fried-Buchalter, 1997), undergraduate entrepreneurs (Sightler and Wilson, 2001), engineering students (French et al., 2008), medical, dental, nursing, and pharmacy students (Henning et al., 1998), and residents in family medicine (Oriel et al., 2004) and internal medicine (Legassie et al., 2008), empirical research on the IP in the context of career development is only beginning to emerge and remains sparse. To the best of our knowledge, only two studies have focused on the preconditions for and specific negative effects of the IP on occupational attitudes (Jöstl et al., 2012; Vergauwe et al., 2015). Given that impostor feelings are also thought to have an impairing effect on the career development process (de Vries, 2005; Vergauwe et al., 2015), further empirical research is needed. Referring to career construction theory, the IP might function as a career theme (Savickas, 2011). A career theme is a controlling implicit idea or perspective “that imposes personal meaning on past memories, present experiences and future aspirations” (Savickas, 2011, p. 26). As such career themes are particularly important in career construction, e.g., in terms of satisfaction or frustration (Savickas, 2011), a closer look at the IP in the career context is strongly required. Hence, the aim of the present investigation was to gain insight into the IP as a potential psychological barrier in the career development process.

**FIGURE 1:**
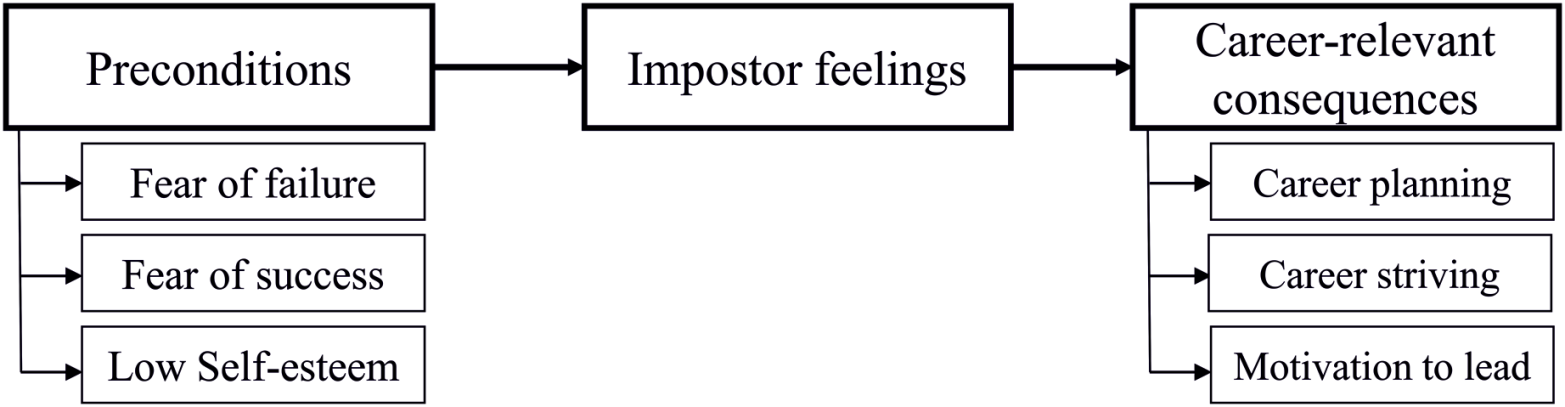
**Hypothesized model of preconditions for and career-relevant consequences of impostor feelings**.

We propose and empirically evaluate a model of the preconditions for the IP as well as its negative consequences for career development (see **Figure 1**). To gain more knowledge about the relation of impostor feelings to aspects of career development, it is essential to empirically examine theoretical accounts of the importance of the IP in career development–especially because it is assumed to have a negative impact (e.g., de Vries, 2005). As the IP is seen as an integrative concept that includes different cognitive features, we decided to focus on three in particular, namely, the fear of failure, the fear of success, and low self-esteem. To explore the consequences of the IP for career development, we investigated the impact of the IP on planning a career, striving for a higher position, and the motivation to take on a leadership role.

## Theoretical Development and Hypotheses

An important aspect of the IP is that it is an integrative concept that is influenced by different motivational constructs. According to the original description of the IP (Clance and Imes, 1978), impostor feelings develop based on a person’s learning history, starting in childhood, in terms of developmental lessons of correlation and causality. When impostors are successful, they attribute it to factors other than ability, such as some kind of luck or charm or knowing the right people (Clance and O’Toole, 1988; Cozzarelli and Major, 1990). Therefore they do not internalize their achievements and remain fearful of failing the next time (see also “the impostor cycle”; Clance, 1985). Further, impostors were shown to feel affectively worse and to suffer a greater loss in state self-esteem than non-impostors if they subjectively failed in an exam (Cozzarelli and Major, 1990). Additionally, they overgeneralize the implications of single failure experiences for their global self-concept (Thompson et al., 1998). They think they are faking being competent and capable (Leary et al., 2000), thereby inhibiting any actual growth in self-esteem (e.g., Cozzarelli and Major, 1990). Studies have shown that impostors have lower performance expectancies (Cozzarelli and Major, 1990) and lower self-perceptions of ability (Leary et al., 2000) than non-impostors. Further, they are unable to acknowledge praise and good performance and are afraid of being rejected if it becomes obvious that they are less capable than they seem (Clance and Imes, 1978).

According to Clance and O’Toole (1988), what drives the negative attitudes described above is the fear of failure. Impostors also fear rejection stemming from achievements that are perceived as inappropriate for them, for example, because they are female (Clance and Imes, 1978). Denying being successful is used as a coping strategy. It is consequently important to take a closer look at the preconditions that foster the occurrence of impostor feelings, namely the fear of failure, the fear of success, and low self-esteem.

### Fear of Failure

Impostors have been found to be very high on neuroticism and low on conscientiousness (Chae et al., 1995), a pattern that fits with their fear of failure and fear of success. Fear of failure is defined as “a tendency to appraise threat and feel anxious during situations that involve the possibility of failing” (Conroy et al., 2007, p. 239). The IP has been found to be best predicted by fear of failure followed by self-handicapping (Ross et al., 2001). Using fear of failure and a lack of confidence in one’s ability as a framework, Kumar and Jagacinski (2006) documented a relation between ability-avoid achievement goals and the IP.

#### Hypothesis 1a

A person’s fear of failure is positively related to his or her impostor feelings. We expect that the more fear of failure a person has, the more impostor feelings will be reported.

### Fear of Success

In addition to fearing failure, impostors fear losing their connection to other people, as might happen with work colleagues when their success is noticed. For instance, as long as someone stays at a current career level he or she does not have to fear the loss of affection from colleagues that might occur if the one is promoted over them. The underlying fear stems from the belief that being successful will result in dislike and resentment by others and thereby loss of affection and approval (Horney, 1936). To handle the fear of loss of affection, impostors try to deny their success (Clance and O’Toole, 1988) or may even handicap themselves (Ross et al., 2001). Investigating why some individuals adopt educational and career goals that appear inappropriately low given their abilities, Fried-Buchalter (1997) observed that the fear of success was positively related to the IP in marketing managers.

#### Hypothesis 1b

A person’s fear of success is positively related to his or her impostor feelings. Therefore, the more fear of success a person has, the more impostor feelings will be reported.

Interestingly, Fried-Buchalter (1997) did neither find a significant correlation between the fear of failure and the fear of success, nor between the fear of failure and the IP, differently to other studies (Ross et al., 2001; Kumar and Jagacinski, 2006; Jöstl et al., 2012). Whereas the fear of failure refers to avoiding anticipated threatening consequences associated with failure (Conroy et al., 2007), thereby strengthening the motivation to do well and succeed in evaluative situations; the fear of success as it relates to the negative consequences which someone anticipate following success like social rejection (Jöstl et al., 2012; Coe et al., 2014), may strengthen the motivation to fail to protect the social support network. As we find impostors to be in a dilemma between the motivation to succeed as well as to avoid social rejection due to success, and because of previous heterogeneous results, we decided to include both constructs in our examination regarding the preconditions of the IP.

### Low Self-Esteem

Normally, achievements foster the growth of self-esteem. In impostors, a missing internal attribution of successful achievements impairs the development of high self-esteem. Several studies found self-esteem to be negatively correlated with the IP (e.g., Chrisman et al., 1995; Thompson et al., 1998; Sonnak and Towell, 2001). It was shown to be negatively correlated with the IP in students (Chrisman et al., 1995; Thompson et al., 1998; Sonnak and Towell, 2001) as well as in working people (Vergauwe et al., 2015). Research has further established that the IP is highly (negatively) correlated with self-esteem but conceptually and empirically distinct from it (e.g., Chrisman et al., 1995).

#### Hypothesis 1c

A person’s self-esteem is negatively related to his or her impostor feelings. Therefore, people with low levels of self-esteem will report high levels of impostor feelings.

In line with Jöstl et al. (2012), and building up on the findings reported above, we investigate the three variables as preconditions for the IP. As stated in the hypotheses 1a-c, we expect the fear of failure and the fear of success to be positively related and self-esteem to be negatively related to impostor feelings.

Now we shift our focus to the impact of impostor feelings on career development. Organizational, career, and vocational scholars have begun to recognize the existence of impostor feelings, yet empirical research on their function in this domain is sparse. For example, one study investigated—in addition to the already mentioned preconditions—the negative consequences of the IP in doctoral students (Jöstl et al., 2012). The researchers showed that impostor feelings are negatively related to research self-efficacy—an important indicator for successful university careers. Another investigation of staff employees of a university found that those suffering from the IP reported significantly less engagement in organizational citizenship behavior (OCB) and lower levels of affective commitment (Grubb and McDowell, 2012). Negative consequences of these occupational attitudes were also shown in working people outside the university (Vergauwe et al., 2015). The study of Vergauwe et al. (2015) confirmed that employees with stronger impostor feelings show less OCB. Additionally, the employees indicated lower levels of job satisfaction.

Vergauwe et al. (2015) also assumed that impostor tendencies might have an impact on organizational behavior and career attitudes. They argued that impostors show a higher continuance commitment (to remain in their current job) because they do not have other or better alternatives. Impostors might not tap their full potential regarding their career opportunities because they do not recognize their own competences. Impostors, who generally experience less confidence (Bernard et al., 2002) and environmental mastery (September et al., 2001), are theoretically less likely to be able to use their resources in the career-development process, to envision possible career paths through career planning, to develop clear career goals or pursue them, and to believe in their capacity to successfully manage career-related tasks as required in higher positions. Yet how impostor feelings are related to career development has not been clearly addressed, and there is a need for more empirical research on how and to what extent impostor feelings affect this process.

Scholars in organizational psychology (de Vries, 2005; Vergauwe et al., 2015) have proposed that the IP is highly relevant for any person engaged in vocational pursuits, particularly given the intense competition and uncertainty that characterizes the present work and career environment. de Vries (2005) hypothesized that impostors damage their careers by allowing their anxiety to trigger self-handicapping behavior, although empirical research on this effect is needed. In line with the idea that learning experiences reinforce impostor feelings, the IP might affect one’s perception of the degree to which one is able to find plausible routes for obtaining important career goals, as well as the likelihood that one feels able to reach those goals. This might have a negative impact on work hope (Juntunen and Wettersten, 2006). Work hope was shown to correlate positively with career planning (Kenny et al., 2010), career decision-making self-efficacy, and vocational identity (Juntunen and Wettersten, 2006). The ability to plan a career, make career-relevant decisions, and gain confidence are important indicators of career adaptability in adulthood and they are needed for career preparation in students (Savickas, 2002). Many studies have illustrated that these aspects of career development play an important role in various work and career outcomes (e.g., better salaries, higher job positions, and career satisfaction; Abele and Spurk, 2009).

One of the next logical steps in studying the IP is to explore its effects on career development explicitly as it was supposed theoretically previously (de Vries, 2005; Vergauwe et al., 2015). By focusing on the career-relevant consequences of impostor feelings, we extend the existing research on the IP by relating it to the career-development process. Specifically, we address how impostor feelings are related to three specific aspects of career development: career planning, career striving, and the motivation to lead.

### Career Planning

In line with Savickas’s (2002, p. 167) statement that one who does not feel safe in daily life is more concerned with “surviving the present than planning tomorrow,” the IP is predestinated to be negatively related to career planning, a variable that includes proactive designing of career plans, which is influenced by the locus of control (Aryee and Debrah, 1992). Impostors were already shown to have an external locus of control (Sightler and Wilson, 2001). Generally, career planning refers to future-orientated thinking and imagining possible pathways to achieve career goals. Moreover, this kind of thinking and envisioning one’s potential future work self has been shown to foster proactive motivation (Strauss et al., 2012). Career planning is one of the most influential aspects of successful career development (Gould, 1979; Orpen, 1994; May, 2005). In line with the assumption that impostors show higher continuance commitment because they do not see better alternatives, even if they are unsatisfied in their current position (Vergauwe et al., 2015), they will not have a strategy or plan for their career because they are unaware of their abilities and therewith any related job opportunities. For instance, if someone is even not aware of the competences he or she possesses in the current position. How, then, can he or she plan to have another even higher position?

#### Hypothesis 2a

People’s impostor feelings are expected to be negatively related to career planning. The more impostor feelings a person has, the less career planning will be report by this person.

### Motivation to Lead

Human resource managers are motivated to include their high-potential and most talented employees in a well-designed succession plan (Parkman and Beard, 2008). They expect such workers to climb the career ladder and to obtain a leadership position. For the plan to work, the employees in question must have the same goal. The underlying construct is the motivation to lead, which is defined as an “individual differences construct that affects a leader’s or leader-to-be’s decision to assume leadership training, roles, and responsibilities and that affect[s] his or her intensity of effort at leading and persistence as a leader” (Chan and Drasgow, 2001, p. 482). These individual differences are assumed to interact with a person’s vocational interests in obtaining a leadership role within a specific domain of work.

People, who like to lead show high affective-identity motivation to lead, see themselves as having leadership qualities, and value competition and achievement. They are extroverted and open to new experiences. Further, they feel confident in their own leadership qualities and exhibit high self-efficacy (Chan and Drasgow, 2001). The so-called can-do factor of a motivational state arises from the perception of self-efficacy, control, and (low) cost (Parker et al., 2010). Impostors, in contrast, are introverted types (Chae et al., 1995; Ross et al., 2001) who perceive little confidence (Bernard et al., 2002) and low levels of career-relevant self-efficacy (Jöstl et al., 2012). Obtaining a leadership position means adapting to new role expectations and managing new challenges, activities that might be avoided by impostors (Klinkhammer and Saul-Soprun, 2009). Instead they will handicap themselves (Ross et al., 2001) by seeking employment elsewhere rather than risk being discovered as frauds when the pressure to advance in position and responsibilities arises and they are expected to seek a more visible role (Parkman and Beard, 2008). “Assessment of the succession plan process has led to the recognition that the IP may play a part in the retention overall and more significantly the derailing of promising candidates in the leadership pipeline” (Parkman and Beard, 2008, p. 30). Even if impostors obviously have the required abilities they will not be motivated to strive for a leadership position because they do not feel confident enough to actualize it. We propose that the IP is an important construct for understanding individual differences in the motivation to obtain a leadership position.

#### Hypothesis 2b

People’s impostor feelings are expected to be negatively related to the motivation to lead. The more impostor feelings one has, the less motivation to lead will be reported.

### Career Striving

In the current career environment that demands high personal motivation to climb the career ladder, proactive career behaviors have gained importance (Parker and Collins, 2010). Empirical research has confirmed that career initiative is positively related to objective career success (Fuller and Marler, 2009). Yet impostors, lacking confidence in their own abilities (e.g., Kumar and Jagacinski, 2006), are most likely to have negative perceptions of their ability to perform well and succeed in the work domain. They seem to have no hope of mastering proactive goal pursuit (Snyder, 2002). Researchers have also found negative relations between impostor feelings and the perception of environmental mastery (September et al., 2001), performance expectancies (Cozzarelli and Major, 1990), and self-perceptions of ability (Leary et al., 2000). Moreover, research suggests that impostors, who lack career-relevant self-efficacy (Jöstl et al., 2012) and environmental mastery, are blocked from striving for higher career levels. Self-efficacy beliefs are a part of human agency, which is important for performance and the active pursuit of valued goals (e.g., Bandura, 2006). Theoretically the IP is an important factor in human performance because it might decrease a person’s motivation to pursue goals or persist in goal pursuit, and it makes sufferers vulnerable in the face of failure.

Hence, we assume that impostor feelings are an important factor in explaining individual differences in career goal attainment. It is reasonable to suppose that the IP is negatively related to proactive career goal pursuit because impostors do not perceive pathways to those goals or to have the ability to reach them. However, research to date has not investigated this relationship specifically.

#### Hypothesis 2c

People’s impostor feelings are expected to be negatively related to career striving. We hypothesize that impostor feelings will decrease striving for higher career stages.

In line with the impostor profile, these avoiding behaviors might not be observable to others in the environment, such as supervisors, peers, or family members. Impostors desire to present a positive impression of themselves to others (impression management) and display perfection in public (Ferrari and Thompson, 2006). Consequently, we assume that impostors will try to protect their image as a career-striving high performer and will therefore keep their doubts to themselves. Thus, it might be possible to distinguish between observable and non-observable career striving. If an employee is asked about a promotion in front of management, he will display keen interest, for example, by taking the information sheet about the job offer with him when everyone can see him do so (observable situation). On his own again (non-observable situation), he will decide to reject the job offer. Thus we expect impostors to show lower career striving in non-observable situations and higher career striving in observable situations.

#### Hypothesis 2d

People’s impostor feelings are expected to be positively related to observable career striving. The more impostor feelings one has, the more observable career striving will be recognizable.

According to career construction theory (Savickas, 2002), the process of vocational development consists of a maxicycle of career stages characterized as periods of growth, exploration, establishment, management, and disengagement. Each stage has its own developmental tasks, such as planning a career in the exploration stage or advancing to new responsibilities, for example, as a leader in the establishment stage. To explore the role of the IP in the career developmental process it is especially interesting to measure its career-relevant consequences for people at different career stages. To this end we tested our hypothesized model (see **Figure 1**) in two samples, namely, university students (Study 1) and working professionals (Study 2).

To summarize, we sought (1) to investigate the relation of the three preconditions fear of failure, fear of success, and self-esteem and impostor feelings and (2) to examine if impostor feelings are negatively related to the aspects of career development career planning, non-observable career striving, and the motivation to lead as well as positively related to observable career striving. (3) As said before, we explored these relations with two samples at different career stages—students and working professionals. To test the preconditions for the IP as well as its consequences, we decided to put all variables into a path model. To examine if one of the consequences might be affected in particular, we looked at the career-relevant consequences with separate path models. We start with Study 1, testing our model in a sample of university students.

## Study 1

We developed two sets of hypotheses, about the preconditions (Hypothesis 1) and career-related consequences (Hypothesis 2) of the IP. In terms of preconditions, we expected impostor feelings to be positively related to fear of failure (Hypothesis 1a) and fear of success (Hypothesis 1b) and negatively related to self-esteem (Hypothesis 1c) in the first study. As students have to pass frequent exams, the fear of failure might play a prominent role in this sample. Even if they succeed very well, if they have impostor feelings, they will not have growth in self-esteem. In turn, this low self-esteem may also be an important predictor for impostor feelings. Fear of success may be less important, as the underlying fear of rejection due to success may be less salient in students, who do not have to compete for promotions.

According to career construction theory (Savickas, 2002), a student sample can be classified as being at the stage of exploration (mainly occurring in 14- to 24-year-olds). The main issue of this stage is to acquire information about occupations in order to make the matching choices that construct a career (Savickas, 2002). Thus, proactive designing of career plans is required, which will be measured as career planning (Aryee and Debrah, 1992). Even if the IP is seen to affect all aspects of career development negatively (Hypothesis 2), special attention should be paid for the influence on career planning (Hypothesis 2a) as a central aspect in students’ career development. The mentioned career choices may also include deciding to apply for offered job positions. In this case, students have to decide how much responsibility they think to be able to handle. As the IP is negatively related to this (September et al., 2001; Bernard et al., 2002), it is hypothesized to reduce non-observable career striving (Hypothesis 2c) as well as the motivation to obtain a leading position (Hypothesis 2b) as it will be the case if they decide to apply for a traineeship. On the other hand, the IP forces them not to show being less capable, thereby fighting against being uncovered as an impostor (Clance and Imes, 1978). Consequently and in line with the assumptions above, the presence of another person who might blow the impostor’s cover, so to speak, will lead to an increase in observable career striving. Therefore, the more impostor feelings an affected student has, the more he or she may pretend to engage in career striving (i.e., the higher their observable career striving will be; Hypothesis 2d).

### Method

#### Participants and Procedure

The sample consisted of 212 university students (70% female) at a European university. The mean age of the sample was 23.23 years (*SD* = 5.36). The largest proportion of the participants were psychology students (78%). At the time of the study, participants’ mean length of study at the university was 4.40 semesters (*SD* = 3.40). We conducted the study in the course of an empirical seminar in the department of economic and organizational psychology (EOP). Participants were recruited via web or by personal contact and invited to be part of a study in the EOP laboratory. The study was approved by the ethical board of the University of Salzburg and carried out in accordance with their recommendations. All students gave informed consent in accordance with the ethical standards of the American Psychological Association (APA). The students were asked to fill out a questionnaire on one of four personal computers and were informed about the voluntary nature of participation and the confidential use of data. They were further informed that there was no right or wrong answers to the questions, and that drawing any personal reference from it would not be possible. In order to assure anonymity, participants were not asked for information that allows inferences to the participants (e.g., names) in the questionnaire. Participants were free to withdraw at any time. Participants were also provided with the name and e-mail address of the responsible investigator. At the end of the questionnaire, participants were thanked for their participation and provided with contact details if they wished to address any questions about the purpose of the study. Filling out the questionnaire took on average 14 min, 10 s (median: 13 min, 14 s). If the participants were psychology students they could receive course credit for participating. Furthermore all participants had the opportunity to win one of three Amazon coupons (3 × €30) if they entered their e-mail address on a sheet of paper on a table in front of the entrance of the EOP laboratory, where some flyers (for measuring observable career striving) were offered, as well.

#### Measures

Preliminary, the items of the preconditions were exploratory factor analyzed using SPSS (Version 22) to explore if they should be insert as three distinct factors in further analysis (see Appendix). Cronbach’s alpha estimates, means, standard deviations, and correlations between measures are reported in **Table 1.** Unless otherwise stated, the measures used a 5-point scale ranging from 1 (*not at all true*) to 5 (*very true*).

**Table 1:** Means, standard deviations, and correlations for the main variables in Study 1.

Variable	*M*	*SD*	1	2	3	4	5	6	7	8
(1) Impostor phenomenon	2.73	0.58	(0.88)							
(2) Fear of failure	3.06	0.82	0.64^∗∗∗^	(0.87)						
(3) Fear of success	2.03	0.61	0.52^∗∗∗^	0.55^∗∗∗^	(0.78)					
(4) Self-esteem	3.82	0.67	-0.71^∗∗∗^	-0.54^∗∗∗^	-0.35^∗∗∗^	(0.89)				
(5) Career planning	2.52	0.51	-0.23^∗∗^	-0.12	-0.11	0.20^∗∗^	(0.87)			
(6) NO career striving	3.63	0.80	-0.12	-0.28^∗∗∗^	-0.15^∗^	0.23^∗∗^	0.03	–		
(7) O career striving	2.55	2.05	0.14^∗^	0.15^∗^	0.05	-0.04	-0.33^∗∗∗^	0.13	–	
(8) Motivation to lead	3.14	0.83	-0.19^∗∗^	-0.30^∗∗∗^	-0.19^∗∗^	0.26^∗∗∗^	0.12	0.55^∗∗∗^	-0.01	(0.94)


**N* = 212 (70% female). NO, nonobservable; O, observable. Entries in parentheses in diagonal are Cronbach’s alpha reliability coefficients. ^∗^*p* < 0.05, ^∗∗^*p* < 0.01, ^∗∗∗^*p* < 0.001.*

##### Impostor feelings

The impostor feelings were assessed using the German translation of the Clance Impostor Phenomenon Scale (CIPS; Clance, 1986; Klinkhammer and Saul-Soprun, 2009). The scale is under copyright by Pauline Rose Clance, Ph.D., from whom permission to copy and use was obtained. It comprised 20 items (e.g., “I can give the impression that I’m more competent than I really am.”).

##### Fear of failure and fear of success

Fear of failure and fear of success were measured using 4 of 24 vignettes developed in the GEHFEM (Bauer et al., 2002; Peters-Häderle, 2006). The vignettes were selected because they describe true-to-life scenarios in the work context. Each vignette consisted of the description of a scenario (e.g., “You will be proposed for a promotion at your workplace. Even if you expected this promotion, you’re insecure, because you don’t know what requirements will be requested. You think:”) and four items. Two of the four items measured fear of failure (e.g., “I hope I do not fail to have the necessary requirements.”), and the other two items measured fear of success (e.g., “Will my colleagues reject me if I climb the career ladder?”). The other scenarios dealt with meeting for a performance evaluation, answering questions in a seminar, and submitting a job application. One item was deleted on the basis of the factor analysis (see Appendix).

##### Self-esteem

We measured self-esteem with the German version of the Rosenberg Self-Esteem Scale (Rosenberg, 1965; Ferring and Filipp, 1996). The scale comprised 10 items (e.g., “On the whole, I am satisfied with myself.”).

##### Career planning

We measured career planning with the German version of Gould’s Career Management Scale (Gould, 1979; Rowold, 2004). The scale comprises nine items. Six items were used for measuring career planning (e.g., “I have a strategy to achieve my career goals.”).

##### Nonobservable (NO) career striving

To measure NO career striving, we presented a pyramid showing several career levels of a potential internship. The positions ranged from 1 (*Staff*) to 5 (*Management*). Each career level included a small description of the tasks and the level of responsibility of the internship at this career level. To ensure the pyramid was understood properly the students rated the benefits and demands of the internships additionally. The internship offers were introduced as a general job board, ensuring that the major of study does not matter. The level descriptions were designed building up on interviews with students concerning internships and possible areas of responsibility. The introductory sentences as well as the pyramid including the descriptions of the internship levels can be found in the Appendix.

##### Observable (O) career striving

To measure observable career striving, we made available fictitious flyers with the pyramid showing the potential career levels on the front. We made five versions of the flyer, highlighting one level of the pyramid on each. Therefore, five stacks of flyers—ranging from 1 (*Staff*) to 5 (*Management*)—were offered to the students. On the back of the flyer participants were debriefed about the fictitious nature of it. The flyers were positioned on a table in front of the entrance to the EOP laboratory. The investigator pointed out that they could take a flyer for further information about the internships with the words “If you would like to get further information about the internships, you can take a flyer with you.” The flyer number was recorded by the investigator after a participant took a flyer with him or her. Flyers wore coded from 1 (*Staff*) to 5 (*Management*). If a participant didn’t take a flyer it was coded with 0 and if he or she took more than one flyer, the highest number was coded because we intended to measure the highest possible career striving one would present.

##### Motivation to lead

The general motivation to lead was requested, thereby entering with the instruction of the HMLI (Hamburg Motivation to Lead Inventory; Felfe et al., 2012): “Regardless of whether you have already collected a lot or a few professional experiences with the leadership of groups, there are several areas (school clubs, leisure etc.) in which the leadership of groups may play a role. The following statements therefore deal with the issue of leadership and responsibility.” One direct-request item was formulated to measure if participants would feel confident taking a leadership position (“I feel confident taking a leadership role.”) and nine more items were taken from the standardized questionnaire for measuring motivation to lead (HMLI; Felfe et al., 2012). An example item of the HMLI is: “I would really be in my element if I took a leadership position.”

##### Demographic variables

Participants reported their sex, age, area of study, university and length of study at the university^1^.

### Analytic Strategy

The conceptual model illustrated in **Figure 1** was tested using path modeling procedures. Descriptive statistics were calculated and analyses were carried out using SPSS and AMOS (Version 22). The most common fit indices that are recommended when reporting path analyses include the Bentler comparative fit index (CFI; Bentler, 1990), and the Steiger-Lind root mean square error of approximation (RMSEA; Steiger, 1990) with its 90% confidence interval. A number of goodness of fit index values are calculated by AMOS; however four goodness-of-fit indices were used to evaluate the path models: the Goodness of Fit Index (GFI), the CFI, RMSEA, and the standardized root mean residual (SRMR). Conventional guidelines were followed: Acceptable model fit is indicated by a CFI and GFI value of at least 0.90, and by a maximum RMSEA and SRMR value of 0.08 (Kline, 2011). For model comparison, the Akaike information criterion and Bayesian information criterion scores are reported.

### Results

The bivariate correlations reported in **Table 1** confirm that impostor feelings were highly correlated with all three preconditions. Fear of failure and fear of success were highly significantly positively correlated and self-esteem was negatively correlated with impostor feelings (*p*s < 0.001). Regarding the associations between impostor feelings and the aspects of career development, significant relations were found for career planning (*p* = 0.001) and the motivation to lead (*p* = 0.005). The expected negative correlation with NO career striving failed to reach significance (*p* > 0.05). In line with our assumptions, we found a significant positive relation with observable career striving (*p* = 0.048).

First, we put all the motivational constructs as well as the aspects of career development into one comprehensive model (see **Table 2**, Path Model 1). Impostor feelings were regressed on fear of failure, fear of success, and self-esteem. Career planning, NO and observable career striving, and the motivation to lead were regressed on impostor feelings.

**Table 2:** Model fit indices and model comparison for the path models in Study 1.

Path model				Model fit				Model comparison		
						
	*χ*^2^	*df*	*p*	CFI	GFI	RMSEA	90% CI RMSEA	SRMR	AIC	BIC
All	137.26	18	0.000	0.759	0.865	0.177	[0.150, 0.205]	0.1213	173.26	233.68
Career planning	0.94	3	0.815	1.000	0.998	0.000	[0.000, 0.071]	0.0112	24.94	65.22
NO career striving	22.68	3	0.000	0.947	0.961	0.176	[0.113, 0.247]	0.0686	46.68	86.95
O career striving	4.81	3	0.187	0.995	0.991	0.053	[0.000, 0.138]	0.0240	28.81	69.09
Motivation to lead	17.64	3	0.001	0.961	0.969	0.152	[0.089, 0.224]	0.0606	41.64	81.92


**N* = 212. AIC, Akaike information criterion; BIC, Bayesian information criterion; CFI, comparative fit index; CI, confidence interval; GFI, goodness of fit index; NO, nonobservable; O, observable; RMSEA, root mean square error of approximation; SRMR, standardized root mean residual. Good model fit is indicated if GFI and CFI ≥ 0.90 and RMSEA and SRMR ≤ 0.08.*

**FIGURE 2:**
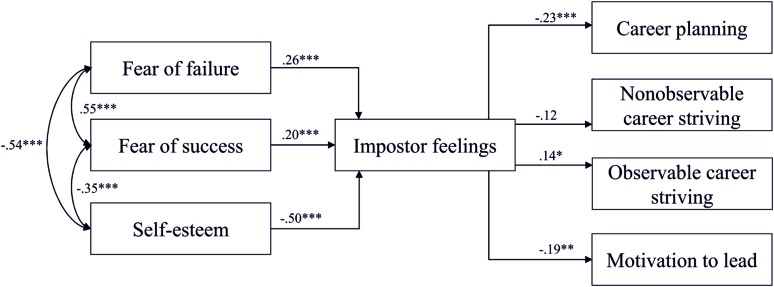
**Results of Path Model 1, displayed with standardized path coefficients.** ^∗^*p* < 0.05, ^∗∗^*p* < 0.01, ^∗∗∗^*p* < 0.001.

As can be seen in **Figure 2**, impostor feelings were predicted by fear of failure (Hypothesis 1a), fear of success (Hypothesis 1b), and self-esteem (Hypothesis 1c), in that participants who reported higher scores on fear of failure and fear of success as well as lower scores on self-esteem reported more impostor feelings (*p*s < 0.001). Together the three preconditions explained 63% of the variance in impostor feelings. Further, impostor feelings were a significant predictor for three of the four aspects of career development. Participants who reported more impostor feelings reported less career planning (*p* < 0.001; Hypothesis 2a) and less motivation to lead (*p* = 0.004; Hypothesis 2b). Hypothesis 2c found only little support in that only a marginal relation to NO career striving was recognized (*p* = 0.083). However, and supporting our assumptions, the more impostor feelings were reported, the higher was the observable career striving (*p* = 0.046; Hypothesis 2d). Six percent of career planning, 12% of NO career striving and 4% of observable career striving, and 12% of the motivation to lead were explained by the variables in the model.

Although impostor feelings were a significant predictor for all three career-relevant consequences, the model including all preconditions and all consequences did not show acceptable model fits (Kline, 2011). As models in which the consequences were included separately (see **Table 2**, Path Models 2–5) were conducted, better fits resulted. The simpler Model 2 only including career planning and Model 5 only including observable career striving showed the best model fits.

### Discussion

We tested a path model that included three preconditions and three career-relevant consequences of impostor feelings in a student sample. Looking first at the preconditions, it becomes obvious that low self-esteem is the most impairing factor followed by high fear of failure and fear of success. This is in line with previous studies that reported high correlations between impostor feelings and self-esteem (e.g., Sonnak and Towell, 2001) and fear of failure in students (Ross et al., 2001). Fueled by the evaluative culture of universities, the prevalent fear of failure is understandable. Students have to pass exams and get good grades to build an optimal foundation for their future career. If students fail to attribute their achievements to internal factors such as stable abilities—if they feel they were lucky this time and fear failing the next time—the fear of failure grows and self-esteem decreases. This process might foster the development of impostor feelings in students (Clance, 1985). The third assumed precondition was also noticeable in students in that the more fear of success was reported, the more impostor feelings occurred.

Although the fear of success is apparent in students, it is assumed to have even more influence in working professionals. As the relation of fear of success and impostor feelings stems from the fear of rejection by people in one’s immediate environment, especially by peers, it is reasonable to suppose that this relation might become even more important in working people, where promotions are rare and highly competitive. Students are generally not in competition for promotions, so they do not necessarily perceive social rejection due to success.

Regarding the consequences of impostor feelings for aspects of career development, career planning was most affected, followed by the motivation to lead. In line with our hypothesis, the more impostor feelings participants reported, the less career planning and motivation to lead they reported. Looking closely at the model fits, it becomes apparent that a model that includes preconditions and career planning fits the data of the student sample best. This is especially alarming because planning might be the most important issue student’s face in their current career-development process. That impostor feelings also inhibit the motivation to lead has consequences for students after they have finished their studies, at which point they may be deciding if they are suited for programs designed to train the leaders of tomorrow. Presumably the more impostor feelings they have, the less likely they will be to pursue such programs. This could have sustainably negative consequences for their career development.

Regarding NO career striving, the negative effect of impostor feelings failed to reach significance. This could either mean that impostor feelings haven’t any negative impact on NO career striving. Or it could indicate that the descriptions of the career levels used to measure this variable did not provide a valid measurement. As the internships offered were only fictitious, it is possible that the students did not have enough information about the positions and the responsibilities they entailed to judge them consistently and comprehensively. This could further affect observable career striving in the way that, they took the flyers with them to use the opportunity to get more information. If they took a flyer they did so in full view of one of the experimenters who stood next to the table. Participants who reported more impostor feelings may have chosen a higher level flyer to maintain their image. As this hypothesis requires confirmation, it would be especially interesting to examine this finding in a worker sample, as well. Unfortunately, this is only possible in a laboratory study and our worker sample was investigated online. To address the problem of too little information about the career levels, we decided to use a worker sample from within one company to base the career levels on the existing organizational structure. Consequently they could choose the position they were striving for in their actual professional life.

## Study 2

In the second study, we wanted to test our postulated model in a sample of working professionals. Regarding the preconditions for impostor feelings, we hypothesized that the fear of success might have more influence in a worker sample where the (for impostors) difficult decision between climbing the career ladder and staying in a harmonious, supportive team is faced more frequently. The fear of rejection by colleagues due to success may in particular foster the occurrence of impostor feelings. Working people who develop impostor feelings may also show a high fear of failure and low self-esteem. Therefore our hypotheses regarding the preconditions (Hypothesis 1) state that impostor feelings should be positively related to fear of success (Hypothesis 1b) and fear of failure (Hypothesis 1a) and negatively related to self-esteem (Hypothesis 1c).

Supporting the assumption that different career-relevant attitudes are salient at different career stages, the postulation of a developmental maxicycle (Savickas, 2002) might give some idea of which attitudes are important at particular career stages. In the domain of career development, research has established that career preparation (e.g., career planning) is an especially important task in emerging adulthood (Super et al., 1996), the age of our student sample. For working adults, career establishment and management variables might be even more important (Savickas, 2002). If an IP sufferer has already entered the labor market it is obvious that the consequences of impostor feelings will become recognizable in the establishment stage (Savickas, 2002). At this stage (typically occurring in 25–44 year-olds), adults might think about their own untapped leadership potential and new responsibilities. Hence, we expected that a model that included the preconditions and career striving (Hypothesis 2c) or the motivation to lead (Hypothesis 2b) as a consequence might fit the data of a worker sample best. Workers are already established in their current job and are assumed to be willing to take the next step. Impostor feelings may function as an inner psychological barrier to this path.

According to career construction theory (Savickas, 2002), the career development maxicycle can be supplemented by a minicycle, where stages such as exploration may occur again when people are in a transition from one career stage to the next as well as each time an individual is destabilized by socioeconomic and personal events. Hence, impostor feelings might have a negative effect on the career-planning process of working professionals, as well (Hypothesis 2a).

### Method

#### Participants and Procedure

The worker sample consisted of 110 employees (50% female) of an international airport in southern Germany. They were recruited from two sectors: sales (*n* = 55) and security (*n* = 55). The mean age was 33.35 years (*SD* = 10.23). The mean working experience was 14.67 years (*SD* = 9.62 years); the mean time of employment in the present company was 7.06 years (*SD* = 5.78 years). Seven participants did not provide any information about their working experience. The study was conducted with the support of the personnel manager. The personnel manager informed the employees about the investigation by e-mail, including a friendly request to participate through a link that directed the employees to an online survey. The study was approved by the ethical board of the University of Salzburg and carried out in accordance with their recommendations. Treatment of the participants was in accordance with the ethical standards of the American Psychological Association (APA). Participants were informed about the voluntary nature of participation and the confidential use of data. They were further informed that there were no right or wrong answers to the questions, and that drawing any personal reference from it would not be possible—neither for the investigators nor for the personnel manager. In order to assure anonymity, participants were not asked for information that allows inferences to the participants (e.g., names) in the questionnaire. Participants were aware that they could withdraw from the online survey at any time. Participants were also provided with the name and e-mail address of the responsible investigator. At the end of the questionnaire, participants were thanked for their participation and provided with contact details if they wished to address any questions about the purpose of the study. Filling out the online survey took on average 15 min, 34 s (median: 13 min, 41 s). The participants did not receive any compensation for their participation in the study.

#### Measures

Preliminary, the items of the preconditions were confirmatory factor analyzed using AMOS (Version 22) to confirm the three distinct factors as explored in Study 1 (see Appendix). Cronbach’s alpha estimates, means, standard deviations, and correlations between measures are reported in **Table 3.**

**Table 3:** Means, standard deviations, and correlations for the main variables in Study 2.

Variable	*M*	*SD*	1	2	3	4	5	6	7
(1) Impostor phenomenon	2.42	0.76	(0.92)						
(2) Fear of failure	3.12	1.09	0.78^∗∗∗^	(0.92)					
(3) Fear of success	2.18	1.01	0.80^∗∗∗^	0.81^∗∗∗^	(0.93)				
(4) Self-esteem^a^	4.56	1.59	-0.76^∗∗∗^	-0.74^∗∗∗^	-0.77^∗∗∗^	–			
(5) Career planning	3.70	1.02	-0.76^∗∗∗^	-0.72^∗∗∗^	-0.74^∗∗∗^	0.80^∗∗∗^	(0.94)		
(6) Career striving	3.03	1.19	-0.51^∗∗∗^	-0.49^∗∗∗^	-0.52^∗∗∗^	0.51^∗∗∗^	0.54^∗∗∗^	–	
(7) Motivation to lead	3.69	1.28	-0.58^∗∗∗^	-0.50^∗∗∗^	-0.52^∗∗∗^	0.55^∗∗∗^	0.59^∗∗∗^	0.66^∗∗∗^	–


**N* = 110 employees (50% female). ^a^Scores ranged from 1 to 7, all other scores ranged from 1 to 5. Entries in parentheses in diagonal are Cronbach’s alpha reliability coefficients. ^∗∗∗^*p* < 0.001.*

##### Impostor feelings, fear of failure, fear of success, and career planning

Impostor feelings, fear of failure, fear of success, and career planning were measured as described in Study 1.

##### Self-esteem and motivation to lead

As the personnel manager advised us to use time economic measurements, we assessed self-esteem with the Single-Item Self-Esteem Scale (SISE; Robins et al., 2001). Robins et al. (2001) established that the SISE and the Rosenberg Self-Esteem Scale show strong convergent validity, thus offering a very economical alternative. The item “I have high self-esteem” was presented with a 7-point scale from 1 (*strongly disagree*) to 7 (*strongly agree*). Furthermore, regarding the motivation to lead, the worker sample answered just the direct-request item as described in Study 1^2^.

##### Career striving

To improve the measurement of career striving, we measure this variable in the worker sample with a pyramid where the potential occupational levels match the company’s organizational levels (Von Vever, 2012). Participants were asked to decide which level in the company they would like to achieve. They were instructed to choose any position regardless of whether anyone else actually held this position. The pyramid showed levels ranging from 1 (*Staff*) to 5 (*Top Management*). Participants were asked about what level they desired to achieve in 2 as well as in 5 years. The 5-year score was used in the analyses to measure the highest possible career striving.

##### Demographic variables

Participants reported their sex, age, employment sector, position, family status, graduation, work experience, language skills, and some general questions regarding working time.

### Results

The bivariate correlations reported in **Table 3** confirm that impostor feelings were highly correlated with all three preconditions in the worker sample, as well. Fear of success was followed by fear of failure this time. Both were highly significantly positively correlated, while self-esteem was negatively correlated with impostor feelings (*p*s < 0.001). Regarding the associations between impostor feelings and the aspects of career development, significant relations were found for career planning, career striving, and the motivation to lead, as well (*p*s < 0.001).

**FIGURE 3:**
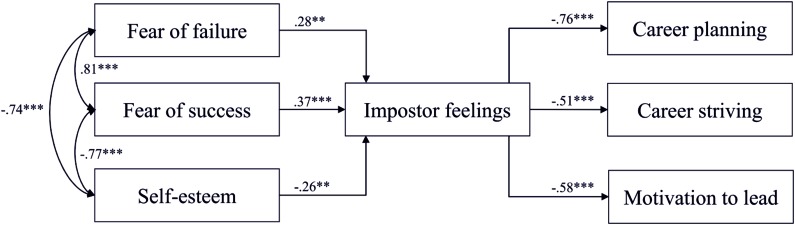
**Results of Path Model 1, displayed with standardized path coefficients.** ^∗∗^*p* < 0.01, ^∗∗∗^*p* < 0.001.

As can be seen in **Figure 3**, impostor feelings were predicted by fear of success (Hypothesis 1b), fear of failure (Hypothesis 1a), and self-esteem (Hypothesis 1c), in that participants who reported higher scores on fear of failure and fear of success as well as lower scores on self-esteem reported more impostor feelings (*p*s < 0.01). Together the three preconditions explained 71% of the variance of the IP. Further, the more impostor feelings were reported, the less career planning participants engaged in and the less career striving and motivation to lead were apparent (*p*s < 0.001). Seventy percent of career planning, 31% of career striving, and 37% of the motivation to lead were explained by the variables in the model.

Even though impostor feelings were a significant predictor for all three aspects of career development, the model including all preconditions and all consequences did not show acceptable model fits, again (see **Table 4**, Path Model 1). Although, impostor feelings affected career planning most negatively (Hypothesis 2a), testing models in which the aspects of career development were included separately (see **Table 4**, Path Models 2–4) showed that the simpler Model 4 that included just the motivation to lead had the best fit in the worker sample (Hypothesis 2b), followed by Model 3, which included career striving (Hypothesis 2c).

**Table 4:** Model fit indices and model comparison for the path models in Study 2.

Model				Model fit				Model comparison		
						
	χ^2^	*df*	*p*	CFI	GFI	RMSEA	90% CI RMSEA	SRMR	AIC	BIC
All	83.93	12	0.000	0.880	0.814	0.234	[0.189, 0.283]	0.1036	115.93	159.13
Career planning	36.98	3	0.000	0.930	0.897	0.322	[0.234, 0.419]	0.0729	60.98	93.38
Career striving	7.30	3	0.063	0.989	0.975	0.115	[0.000, 0.223]	0.0476	31.30	63.70
Motivation to lead	4.76	3	0.191	0.996	0.983	0.073	[0.000, 0.191]	0.0340	28.76	61.16


**N* = 110. AIC, Akaike information criterion; BIC, Bayesian information criterion; CFI, comparative fit index; CI, confidence interval; GFI, goodness of fit index; NO, nonobservable; O, observable; RMSEA, root mean square error of approximation; SRMR, standardized root mean residual. Good model fit is indicated if GFI and CFI ≥ 0.90 and RMSEA and SRMR ≤ 0.08.*

### Discussion

In the second study, we tested a model that included preconditions (fear of success, fear of failure, self-esteem) and consequences (career planning, career striving, motivation to lead) of impostor feelings in a sample of workers. As expected, fear of success became more important and was therefore the strongest predictor of impostor feelings in the worker sample. This supports the assumption that impostor feelings are developed as a coping strategy to deal with the fear of being rejected by work colleagues, for instance, in the event one is promoted. As long as the supposed high achievers deny being successful at all, they do not have to fear rejection by envious colleagues. A high fear of failure and low self-esteem were also very strong and nearly equally important predictors, again supporting the close relation of these constructs and the IP. The preconditions led to an increase in impostor feelings, which in turn led to a decrease in aspects of career development.

Looking at the single components, impostor feelings affected career planning most negatively. As suggested by the minicycle of career construction theory (Savickas, 2002), career planning played an important role in this sample, as well. A highly promising career plan may include climbing the career ladder within or outside the current company. As impostor feelings develop from low self-esteem and a high fear of failure or success, participants suffering from the IP may not have seen ways to take the next steps, thereby showing less clear plans or strategies for their career. This assumption is further supported in that the more impostor feelings participants reported, the less career striving and motivation to lead they reported. Thinking about the fear of failure as a precondition of the IP and the less striving for a higher career level—especially a leadership position—is particularly interesting in light of job market development. Impostors avoid striving because they fear failing in a higher position. Their first priority is to secure a job and maintain the perception that they are good at the job they have.

Looking at the model fits, it becomes clear that a model that includes the preconditions and the motivation to lead fits the data of the worker sample best. People with impostor feelings, despite having entered the labor market successfully will not strive for a leadership position. Even if they have all the required strengths they will not risk being exposed as less capable, which becomes more likely under growing responsibilities. In line with earlier research showing that impostor feelings go along with the fear of failing in public (Ferrari and Thompson, 2006), it is obvious that such people will not be motivated to obtain a leadership position with many chances to fail in public. People may feel that in leadership positions they will be constantly evaluated and reviewed by others, increasing the risk of being exposed as a fraud.

The high influence of fear of success in working people is also of concern. This fear may cause people to self-handicap and sabotage their careers by staying in jobs where they do not realize their full potential. At the top of any organization the positions and the number of people qualified to fill them are limited. Therefore, successful interventions for dealing with impostor fears would serve employees and employers alike.

## General Discussion

This investigation aimed to explore the IP in the career-development context. We tested a model that included preconditions and consequences of impostor feelings to gain an understanding of how the IP develops and impairs (see **Figure 1**). Further, we wanted to explore the IP at different career-development stages. To this end, we tested our model in two samples: university students (Study 1) and working professionals (Study 2). While impostor feelings were most powerfully predicted by low self-esteem and the fear of failure in the student sample (Study 1), the fear of success played the most prominent role and was the strongest predictor in working professionals (Study 2). The findings are in line with earlier studies that reported a relation of impostor feelings and fear of failure in students (Ross et al., 2001) and with fear of success in working people (Fried-Buchalter, 1997). That the fear of success had more influence on impostor feelings in the worker sample can be explained through the fact that they are aware of their colleagues’ opinions and are more affected by their judgments than students are. The relevance of fear of success in the worker sample is in line with the previous finding that workplace social support can act as a buffering variable in the relationships between impostor tendencies and work outcomes (Vergauwe et al., 2015). Vergauwe et al. (2015, p. 578) found that, “when social support is high, the negative relationships between impostor tendencies and satisfaction and OCB disappear.” As long as impostors do not acknowledge their successes, they do not have to face being promoted and losing their workplace social support. If they stay with their team of supportive colleagues, they are more satisfied with their job. Furthermore, they invest extraordinary time and may show great performance without any loss of affection. Regarding self-esteem, we replicated the high negative relation between impostor feelings and self-esteem in students (50% explained variance) as well as in working professionals (58% explained variance). When we put the variables in a path model, it became clear that low self-esteem is a very important precondition of the IP. Several other studies also found self-esteem to be negatively related to impostor feelings but until now only two studies (Jöstl et al., 2012; Vergauwe et al., 2015) have addressed the function of self-esteem as a precondition. Our findings therefore strongly support this hypothesis. Together the preconditions explained large proportions of the variance in impostor feelings—63% in the student sample (Study 1) and as much as 71% in the sample of working professionals (Study 2)—adding support to our suggestion that they play a role as preconditions. But even if they explain large proportions of the IP, we do not believe that the IP can be reduced to low self-esteem and the high fear of failure or being successful. As previous research (Vergauwe et al., 2015), we understand the IP to be a maladaptive phenomenon that incorporates a set of cognitive features. Other variables in addition to the ones examined in the current investigation, such as inappropriate attributional styles, perfectionist concerns, and the fear of being exposed, encourage its appearance. It is the interaction between all these variables that determines the development of the IP as well as its maintenance and reinforcement (see also “the impostor cycle”; Clance, 1985).

In addition to the preconditions, we were especially interested in how the IP might influence aspects of career development. Our results enhance the current literature by showing that impostor feelings have not only preconditions but also negative consequences in the context of career development. In both samples impostor feelings especially impaired the career-planning component, meaning those with more impostor feelings had less clear plans and fewer strategies for their career. The key to this finding can be found in the description of the IP. As people with impostor feelings are not aware of their competences, it is understandable that they do not have a great vision for their future career. Together with low self-esteem and the fear of failure or success, the IP is a real barrier to a successful career plan. Many high-potential workers may be lost during the career-development process because they do not take the right steps to recognize their own potential and are certain that they are just pretending to be capable, anyway. With regard to career striving, we found a differentiation of NO and observable career-striving measures in that impostor feelings related to them in a negative and a positive way, respectively. We therefore recommend future investigators to take this differentiation into account. Our results show that the IP had a strong influence on career development in the worker sample (Study 2)—even more than in the student sample (Study 1). This is particularly interesting because it suggests that the negative consequences of the IP may even increase over the career-development process. This should be further investigated in future research, as well. As impostor feelings make it hard to plan a career, for students as well as for working professionals, it is particularly important to take the IP into account when thinking about career-development processes. Working impostors seem to act under the slogan “never change a running system” and avoid pursuing higher career levels within their organization. Further they do not feel capable of obtaining a leadership position. As they score low on self-esteem and high on the fear of failure, they probably fear the expected challenges and are convinced that they would not manage a change successfully. Additionally and indicated by the high fear of success, they do not push their career out of fear of rejection by colleagues. Consequently and in line with earlier research (Ross et al., 2001), it can be expected that they are handicapping themselves on their career path. Regarding succession planning, previous theoretical assumptions (Parkman and Beard, 2008) receive some support from the findings of our studies. Promising candidates might seek employment elsewhere rather than risk being discovered as less capable in a higher position—especially a visible leadership role.

### Limitations, Strengths, and Future Research

Finally, this study also has some limitations. First, we used a cross-sectional research design, which does not allow drawing solid causal conclusions regarding the observed associations. Following earlier hypotheses (Vergauwe et al., 2015), the basis for assuming that impostor feelings influence aspects of career development and not vice versa comes from the fact that, for example, career planning is more directly related to concrete actions (e.g., actualizing a career plan) and therefore serves as a predictor of successful career development (e.g., May, 2005), whereas impostor feelings are more closely linked to more stable constructs such as depression or general anxiety (e.g., Bernard et al., 2002). Nevertheless, future research should add support for the hypotheses by using a longitudinal design and observing actual career development. This could be realized by including IP measures in employee surveys or panel data collection.

Second, most of the variables were measured using self-reports, which forces us to consider common method bias. In particular, if having impostor feelings implies a tendency to downgrade oneself, some findings could partially reflect underreporting effects (Vergauwe et al., 2015). Therefore, the difference between NO and observable career striving could be explained not only through the mechanisms discussed earlier in this paper but also by underreporting effects. To empirically distinguish between underreporting effects and true findings, future research should combine NO and observable measures. This could even expand and confirm the results reported here.

Furthermore, even if large proportions of the variance in the career relevant variables could be explained in the worker sample, the explained variance in the student sample was rather small. Therefore, the influence of impostor feelings on additional variables which are relevant for career development, such as career adaptability (Savickas and Porfeli, 2012) or knowledge of job market (Spurk and Volmer, 2013), should be investigated in future research. In line with the positive relationship of impostor feelings and observable career striving, it is thinkable that the IP is also positively related to career adaptability, as impostors might adapt to changing conditions as far as possible for protecting their image of being highly capable. Moreover, impostors who already entered the labor market could have more knowledge of job market–an attribute positively associated with career success (Spurk and Volmer, 2013)–as they are continually searching for another job opportunity in case of being exposed as frauds. Additionally, it could be useful to address variables such as career planning more detailed. As students’ career plans are supposed to detail exploration in breadth, plans of working professional may call for exploration in depth (Savickas, 2011). It is possible, that the measurement used in our studies mainly addressed exploration in depth. Therefore, the use of different measurements that address both dimensions could be a beneficial addition in future research.

### Practical Implications

The present investigation adds to the current literature on psychological barriers to a successful career-development process by taking the IP, an integrative motivational construct, into account. Our results offer a starting point for useful interventions, in that they provide evidence for three specific preconditions that form a dispositional risk for the development of impostor feelings in students as well as in working professionals. Further, the current investigation demonstrated that impostor feelings can have an impact on aspects of career development, which could, for instance, be enlightening for career counselors. If they do not know why high-achieving employees are not willing to climb the next step on a career ladder they could investigate if impostor feelings are playing an inhibiting role. For example, impostor feelings could be approached by including them in a Career Construction Interview or Career Story Interview (Savickas, 2011) as a career theme (Di Fabio, 2013).

The finding that impostor feelings affect different aspects of career development at different career stages is relevant to general support measures for IP sufferers equally whether they are students or already employees. People affected by strong impostor feelings could especially benefit from individual coaching programs that focus on the reduction of fear of failure or success as well as on the enhancement of self-esteem. Clients should get support in learning how to deal with fears of failure and how to attribute successes to their own abilities, thereby boosting their self-esteem. Given that the fear of success is associated with the fear of rejection by colleagues and that social support could buffer the negative effects of the IP (Vergauwe et al., 2015), it might be expedient to provide networking programs or supervision groups where employees have the chance to share their experiences. Incorporating the IP topic in the support measures might enhance the reduction of impostor feelings as well as their negative effects on the career-development process. A better understanding of the functions of impostor feelings in the career-development process may also contribute to the enhancement of career coaching and human resource development practices as more is understood about how impostor feelings can be reduced in non-clinical populations.

### Conclusion

The results presented in this paper show that a high fear of failure in students—young people under performance pressure—and the fear of rejection by colleagues due to success in working professionals as well as low self-esteem provide strong preconditions for the development of impostor feelings. By connecting the IP with variables relevant for successful career exploration and establishment, namely, career planning, career striving, and the motivation to lead, the first step is done to include this multifaceted variable in the context of career development. This investigation is of high relevance for organizational development (e.g., succession planning) and individual careers, underlining the impact of the IP as a considerable inner barrier to career development.

## Author Contributions

Both authors (MN, ET-M) substantially contributed to the conception and the design of the work as well as in the analysis and interpretation of the data. As the first author (MN) prepared the draft, the second author (ET-M) reviewed it critically and gave important intellectual input. Both (MN, ET-M) worked for the final approval of the version that should be published. Both authors (MN, ET-M) are accountable for all aspects of the work in ensuring that questions related to the accuracy or integrity of any part of the work are appropriately investigated and resolved.

## Conflict of Interest Statement

The authors declare that the research was conducted in the absence of any commercial or financial relationships that could be construed as a potential conflict of interest.

## Footnotes

1 Three short scales for measuring general self-efficacy beliefs (Beierlein et al., 2013) and core self-evaluations (Stumpp et al., 2010) in Study 1, and occupational self-efficacy (Abele et al., 2000) in Study 2, were also investigated to explore their reliability. These scales will be used in another investigation and were not included in further analysis of the current paper.

2 To ensure that the direct-request item measured motivation to lead appropriately, a correlation analysis with the HMLI items was conducted in Study 1, showing a highly significant correlation (*r* = 0.62, *p* < 0.001).
