# Supplementary material for: An Inner Barrier to Career Development: Preconditions of the Impostor Phenomenon and Consequences for Career Development
Source: Front Psychol. 2016 Feb 4;7:48. doi: 10.3389/fpsyg.2016.00048 (PMC4740363; doi:10.3389/fpsyg.2016.00048)
Supplement: Supplementary file 1 [file Data_Sheet_1.PDF]

Appendix

Introductory sentences and the pyramid for measuring non-observable career striving

"In order to complete the study successfully, while being an intern is usually required. In addition, you can make contacts for your career and gain valuable skills and experience. We have put together different types of internships that relate to different areas of responsibility. The higher up an internship is in the pyramid, the more demanding it is and the more you are allowed to take over responsibility. Please chose for which internship you would apply and select this."

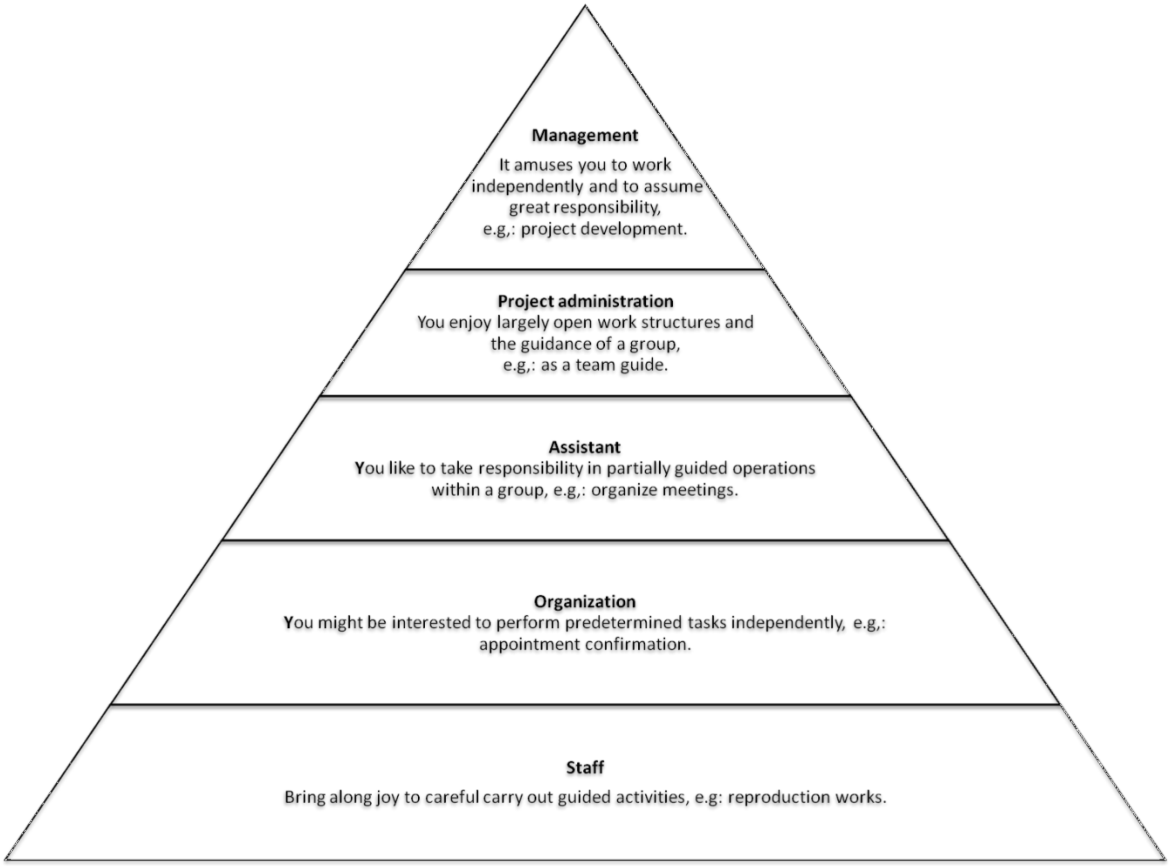

Factor Analyses

The measures for fear of failure, fear of success, and self-esteem were exploratory factor analyzed using a common factor analysis with promax rotation in SPSS (SPSS Inc., 2007) to test whether they are three distinct preconditions (see Table 5). The Kaiser-Meyer-Olkin coefficient was .90, and the Bartlett-Test was significant ($p < .001$). The analysis showed that the three factor solution explains 51% of the variance. One item that was induced to measure fear of success ("Will my new colleagues like me?") loaded most on the fear of failure factor. As the content-based classification does not allow attaching it to this scale, we deleted the item completely in both studies. All other items loaded clearly on the initial factor. Factor 1 comprised 10 items measuring self-esteem (a = .89), Factor 2 comprised 8 items measuring fear of failure (a = .87), and Factor 3 finally comprised 7 items measuring fear of success (a = .78). Consequently, we included the three factors as distinct

preconditions in our path model in Study 1. To test the same factor structure in Study 2, we calculated a confirmatory factor analysis using AMOS 22 (SPSS Inc., 2007). Fear of failure and fear of success were included as unobserved variables. As self-esteem was measured using the single-item self-esteem scale (Robins et al., 2001) it was entered as observed variable in the CFA. The CFI was .90 and the SRMR was .06, thereby indicating acceptable model fit (Kline, 2011). The standardized regression weights are displayed in Table 6.

Table 5

Factor loadings of the EFA with promax rotation in Study 1

Item	Factor		
	1	*2*	*3*
1 SE 10	.80		
2 SE 1	.79		
3 SE 2 (R)	.79		
4 SE 7	.76		
5 SE 9 (R)	.74		
6 SE 3	.72		
7 SE 5 (R)	.68		
8 SE 8 (R)	.63		
9 SE 4	.55		
10 SE 6 (R)	.42		
11 FOF 6		.88	
12 FOF 2		.87	
13 FOF 7		.79	
14 FOF 5		.74	
15 FOF 1		.74	
16 FOF 3		.72	
17 FOF 8		.52	
18 FOF 4		.45	
19 FOS 7		.46	
20 FOS 5			.82
21 FOS 4			.77
22 FOS 1			.72
23 FOS 2			.57
24 FOS 8			.57
25 FOS 3			.56
26 FOS 6			.39

Note. Factor loadings ≥ .39 are displayed.
Items are sorted by scale and size.

Table 6

Factor loadings of the CFA in AMOS in Study 2

Item	Factor	
	1	*2*
1 FOF 5	.94	
2 FOF 1	.83	
3 FOF 6	.81	
4 FOF 3	.78	
5 FOF 2	.70	
6 FOF 7	.69	
7 FOF 4	.66	
8 FOF 8	.62	
9 FOS 3		.87
10 FOS 2		.85
11 FOS 8		.85
12 FOS 1		.83
13 FOS 5		.80
14 FOS 6		.73
15 FOS 4		.70

Note.Items are sorted by scale and size.
